# Device sensitivity and false alarms can reshape regression-to-the-mean in simulated epilepsy trials

**DOI:** 10.64898/2026.07.30.26359361

**Published:** 2026-08-02

**Authors:** Daniel M. Goldenholz, Shira R. Goldenholz, Rohan Bhansali, Ted J. Kaptchuk, Brandon Westover

## Abstract

Automated seizure detection devices are increasingly plausible tools for epilepsy trials, but no device is perfect. We used CHOCOLATES, a realistic seizure diary simulator, to examine how device sensitivity and false alarm rate (FAR) affect regression-to-the-mean (RTM) and placebo median percentage change (MPC) in a simulated randomized trial design. For each device condition, 100,000 potential participants were generated; eligibility was assessed during a 2-month baseline, followed by a 3-month test period. With FAR fixed at 0, reducing sensitivity from 100% to 10% increased the fraction of eligible participants exhibiting RTM from 38.2% to 64.8% and increased placebo MPC from 14.7% to 48.1%. With sensitivity fixed at 100% and expected FAR correction, increasing FAR from 0 to 1 alarm/day increased RTM from 38.2% to 53.2% and placebo MPC from 14.7% to 31.3%. Imperfect seizure detection can therefore change the apparent placebo response expected from RTM.

**Short summary for table of contents:**

In simulated epilepsy trials, imperfect seizure detection altered regression to the mean and placebo median percentage change. Trial planning should model detector sensitivity and false alarm rate before device-derived seizure counts are used as endpoints.

## Full Text Availability

The license terms selected by the author(s) for this preprint version do not permit archiving in PMC. The full text is available from the preprint server.

